# Analysis of the complete genome of hepatitis B virus subgenotype C2 isolate NHB17965 from a HBV infected patient

**DOI:** 10.12688/f1000research.15090.3

**Published:** 2018-09-10

**Authors:** Modhusudon Shaha, Palash Kumar Sarker, Md. Saddam Hossain, Keshob Chandra Das, Munira Jahan, Shuvra Kanti Dey, Shahina Tabassum, Abu Hashem, Md. Salimullah

**Affiliations:** 1 Microbial Biotechnology Division, National Institute of Biotechnology, Dhaka, 1349, Bangladesh; 2 Molecular Biotechnology Division, National Institute of Biotechnology, Dhaka, 1349, Bangladesh; 3 Department of Virology, Bangabandhu Sheikh Mujib Medical University, Dhaka, 1000, Bangladesh; 4 Department of Microbiology, Jahangirnagar University, Dhaka, 1342, Bangladesh

**Keywords:** HBV/C2, Chronic, Non-recombinant, Bangladesh

## Abstract

The burden of chronic hepatitis B virus (HBV) infections is increasingly detected nowadays. Herein, we report a complete genome of HBV subgenotype C2 (HBV/C2) from a HBV infected patient. Complete genome analysis revealed that the isolated strain was a non-recombinant wild type and had several regular substitutions in the reverse transcriptase domain and small surface proteins of HBV. This study may help clinicians and scientists gain in-depth knowledge on the current substitutions of HBV/C2 genome and to identify potential therapies against HBV infections.

## Introduction

The burden of chronic liver disease caused by hepatitis B virus (HBV) is increasingly detected at present ^1^. Globally, more than 2 billion people have been infected by HBV ^2, 3^ and, according to the World Health Organization (WHO) approximately 257 million were living with HBV in 2017. In Bangladesh, the rate of HBV chronicity is 2–6% ^4^, which makes it relatively higher risk than some infectious diseases, for example, Hepatitis C virus ^5^ , Human immunodeficiency virus ^6^.

HBV genome comprises a partially double-stranded covalently closed circular DNA that encodes four highly overlapping major open reading frames ^7^. Due to the absence of proof-reading activity, the mutation rate of HBV is high ^8^, which may induce the possible recombination events of the strains ^9^. Most chronic HBV cases have a high possibility of causing liver cirrhosis ^10^ and hepatocellular carcinoma ^11^. In Bangladesh, there is scarce of complete genome sequence of HBV chronic strain of subgenotype C2. Hence, we isolated the complete genome of a HBV/C2 strain collected from a patient having HBV infection carrying the virus for a long time.

## Methods

### Isolation and sequencing

An HBV-positive plasma sample was collected from a 45-year-old male patient in a tertiary hospital in Dhaka, Bangladesh after obtaining the patient’s written informed consent. The infected patient might have chronic liver disease, as determined by ultrasonography. The patient was diagnosed with the possibility of chronic HBV infection, suggested by the presence of ascitis and enlarged spleen, after the positive reaction of anti-HBc total and with a high viral load in the plasma. However, the patient was not showing signs of jaundice, though was affected by fever, nausea, vomiting and fatigue. The study was approved by the Research Ethics Committee of National Institute of Biotechnology, Bangladesh (NIBREC2015-01). The patient was not taking any antiviral therapy and was diagnosed 1 month prior to obtainment of the plasma sample. HBV DNA was extracted from the sample using the QIAamp MinElute Virus Spin kit (Qiagen, Germany). The complete HBV genome was amplified by six sets of primer pairs used previously in another study ^12^ using a conventional PCR method. The primer sequences and their annealing temperatures were as follows: set 1, forward- AAGCTCTGCTAGATCCCAGAGT, reverse- AGTTGGCGAGAAAGTGAAAGCCTG, 56°C; set 2, forward- CCTATTGATTGGAAAGTATGTCA, reverse- AACAGACCAATTTATGCCTA, 48°C; set 3, forward- GAGACCACCGTGAACGCCCA, reverse- CCTGAGTGCTGTATGGTGAGG, 56°C; set 4, forward- TTCACCTCTGCCTAATCATC, reverse- ATAGGGGCATTTGGTGGTCT, 52°C; set 5, forward- TCAGGCAACTATTGTGGTTTCA, reverse- GGGTTGAAGTCCCAATCTGGATT, 51°C; set 6, forward- GGGTCACCATATTCTTGGGAA, reverse- CGAGTCTAGACTCTGTGGTA, 51°C. For a mixture of 25 µl reaction volume, 12.5 µl of 2X MasterMix (Thermo Fisher Scientific, USA), 1 µl each of forward and reverse primers (IDT, USA), 9.5 µl of nuclease-free water (Thermo Fisher Scientific, USA) and 2 µl of template DNA were used. The condition of the PCR reaction was 1 cycle at 95°C for 10 min, 35 cycles at 95°C for 1 min, with the aforementioned annealing temperatures for 1 min and 72°C for 1 min, and a final cycle for 10 min at 72°C. Sanger sequencing was performed using the BigDye Terminator version 3.1 cycling sequencing kit (Applied Biosystems, USA) by ABI 3130 Genetic Analyser (SeqGen, CA, USA) and by thermal cycler (Sigma-Aldrich, Germany) using the described annealing temperatures as per manufacturer’s instructions after the purification of PCR products using PureLink PCR Purification Kit (Thermo Fisher Scientific, USA), performed in accordance with the manufacturer’s protocol. Next, the sequenced contigs were assembled using the Seqman tool of DNASTAR Lasergene version 7.2 ^13^.

### Analysis

The subgenotyping and mutation analysis of the sequenced genome were performed using the HBV Geno2Pheno tool version 2 ^14^ using the default parameters, comparing against the HBV genotypes consensus sequences. Recombination analysis of the sequence was performed using the NCBI genotyping tool. The complete genome was deposited in the GenBank under the accession number MH220971.

## Results and discussion

**Figure 1.**
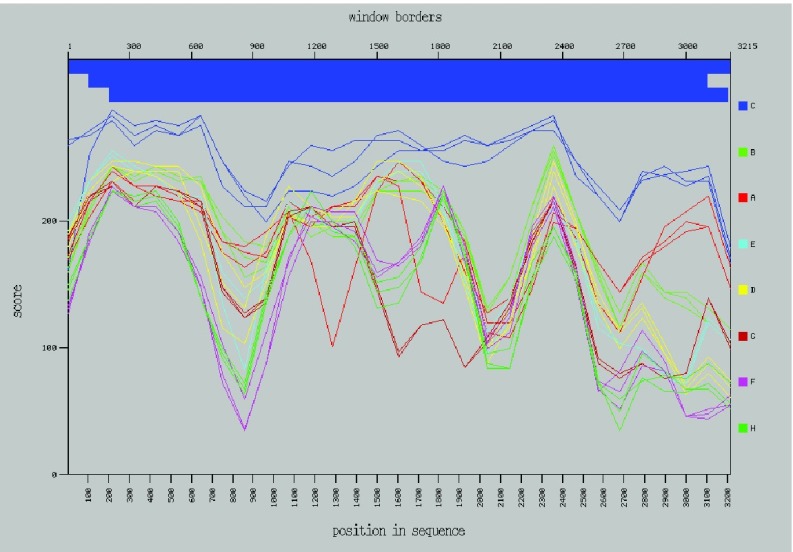
Recombination analysis of the isolate NHB17965. The Simplot diagram was generated using the NCBI Genotyping tool.

Analysis of the complete genome denotes that the isolate studied here, termed NHB17965, comprises HBV genotype C and subgenotype C2 (HBV/C2) with a GC content of 48.77%. Recombination analysis using the NCBI Genotyping tool showed that NHB17965 is a non-recombinant wild-type HBV isolate ( Figure 1).

The patient was diagnosed with chronic HBV infection. Although, the patient was tested positive 1 month prior to obtainment of the plasma sample, he might be infected much earlier as he had a minor surgery few years back and his brother was positive for HBV years ago. Isolate NHB17965 was observed to have amino acid substitutions H9Y, N13H, I91L, P109S, T128N, I269L and V278I in the polymerase domain and S53L, P120T, I126T and S210N in the small hepatitis B surface protein as analysed by HBV Geno2Pheno tool ^14^, compared against the HBV genotypes consensus sequences. These substitutions may be the results of regular genomic changes to HBV because of a lack of proof-reading activity of the viral reverse transcriptase, and may not signify any danger. 

The findings of this study may help clinicians and scientists to gain substantial knowledge about the current genomic substitutions of HBV/C2 and to decide treatments against chronic HBV infections.

## Data availability

Genome of the HBV strain isolated in this study, MH220971.
