# Supplementary material for: Analysis of the complete genome of hepatitis B virus subgenotype C2 isolate NHB17965 from a HBV infected patient
Source: F1000Res. 2018 Sep 10;7:1023. Originally published 2018 Jul 9. [Version 3] doi: 10.12688/f1000research.15090.3 (PMC6124380; doi:10.12688/f1000research.15090.3)
Supplement: Peer review history

# Peer review: Referee response for version 3

**DOI:** 10.5256/f1000research.17752.r38142

**Published:** 2018-09-19

**Authors:** Paul Klapper

**Affiliations:** 1 University of Manchester, Manchester, UK

As outlined in previous reviews of this m/s there is no laboratory evidence that this patient had chronic hepatitis or chronic hepatitis B infection. He was diagnosed  with HBV infection only 1 month prior to the investigation described in the manuscript. 

 At the end of the introduction the authors state the virus was obtained from *"A patient having HBV infection for a long time". *There is no evidence presented for this, he was only diagnosed 1 month prior to investigation. 

 In results and discussion, paragraph 2, we are told *"the patient was diagnosed with chronic HBV infection" *there then follows an attempt to justify this statement.:- he had minor surgery a few years back, his brother was diagnosed with HBV a few years ago *. *This is circumstantial rather than clear proof that this patient had chronic hepatitis.

 It appears that without retesting the patient more than 6 months after initial diagnosis, there is no way that this patient can be said to have chronic hepatitis or chronic HBV infection. 

 Introduction 2nd paragraph " *In Bangladesh, there is scarce of complete genome sequence of HBV chronic strain of sub-genotype C2"* might be better expressed as ' *In Bangladesh there is a lack of complete genome sequences of HBV sub-genotyoe C*' - as outlined above, there appears to be no concrete evidence that the strain of HBV analysed in this paper caused chronic hepatitis.

I have read this submission. I believe that I have an appropriate level of expertise to confirm that it is of an acceptable scientific standard, however I have significant reservations, as outlined above.

# Peer review: Referee response for version 2

**DOI:** 10.5256/f1000research.17379.r37523

**Published:** 2018-09-04

**Authors:** Ashesh Kumar Chowdhury

**Affiliations:** 1 Department of Immunology, BIRDEM General Hospital, Dhaka, Bangladesh

The reporting of a sequence analysis of complete genome of subgenotype C2 of hepatitis B virus is new in Bangladesh perspective. The article as ‘research note’ is quite acceptable.

 But, regarding the ‘chronic status’ of patient, it may be changed at all portion of the article except in ‘results and discussion’ part where it may be kept as a possibility of being chronic (because ‘history of minor surgery few years back and brother was positive for HBV years ago’). The chronic status of the patient was not established by serological evidence (e.g. HBsAg positivity 6 months back).The change of ‘chronic’ status  starts from title- where ‘from a patient with uncomplicated chronicity’ may be changed to ‘HBV infected patient’

 In the abstract, ‘a patient with chronic HBV infection’ may be changed to ‘HBV infected patient’ and from ‘to identify potential therapies against chronic HBV infections’ ‘chronic’ word may be omitted.

 In the introduction part- ‘Most of the HBV cases are chronic, …. hepatocellular carcinoma’ is misleading.  It needs rewriting as ‘Most chronic HBV cases have a high possibility causing liver cirrhosis and hepatocellular carcinoma’. In last line, ‘from a patient with chronic HBV infection carrying the virus for a long time’ may be replaced by ‘from a patient having HBV infection’.

 In the methods part-  ‘The infected patient……..determined by USG. The Patient was diagnosed with chronic HBV infection, suggested by presence of the presence of ascitis…..high viral load in the plasma.’ These two lines need rewriting, mentioning the possibility of being chronic. But, again lack of serological evidence needs to be mentioned.

I have read this submission. I believe that I have an appropriate level of expertise to confirm that it is of an acceptable scientific standard.

# Response

**Published:** 2018-09-06

**Authors:** Modhusudon Shaha

**Affiliations:** National Institute of Biotechnology, Bangladesh

We would like to thank the reviewer for his constructive comments on the manuscript. Herein, the responses to the reviewer's comments are given.

 From the title- ‘from a patient with uncomplicated chronicity’ is changed to ‘HBV infected patient’. 

 The abstract is rewritten considering the valuable suggestions of the reviewer. The introduction part is revised according to the reviewer's comments. The commented sentences are revised in the methods section.

# Peer review: Referee response for version 2

**DOI:** 10.5256/f1000research.17379.r37107

**Published:** 2018-08-21

**Authors:** Paul Klapper

**Affiliations:** 1 University of Manchester, Manchester, UK

The authors have made a reasonable attempt to address the points raised in my previous review of this manuscript however a fundamental concern remains. There is no laboratory evidence that this patient had chronic HBV infection, there is only evidence that the patient had chronic hepatitis and that hepatitis B infection was detected at the point the patient was investigated.

 It is not possible to say that the patient had chronic hepatitis B virus infection.

 Abstract: *“Hence, we isolated the complete genome of a HBV/C2 strain collected from a patient with chronic HBV infection carrying the virus for a long time”.*

 Yet the text of the paper does not support the statement of virus carriage for a long time, in methods we find:

*"The infected patient had chronic liver disease, as determined by ultrasonography. The patient was diagnosed with chronic HBV infection, suggested by the presence of ascitis and enlarged spleen, after the positive reaction of anti-HBc total and with a high viral load in the plasma"*

 and in Results/Discussion we find:

*"The patient was diagnosed with chronic HBV infection. Although, the patient was tested positive 1 month prior to obtainment of the plasma sample, he might be infected much earlier as he had a minor surgery few years back and his brother was positive for HBV years ago"*

 These findings confirm that the patient had chronic hepatitis they do not confirm that the patient had chronic hepatitis B infection. As previously outlined, the standard definition of chronic hepatitis B infection is the detection of hepatitis B surface antigen in serum for more than 6 months. As there is no laboratory evidence of carriage of hepatitis B virus surface antigen for more than 6 months it is only possible to say this patient had chronic hepatitis and suggest that it is likely to represent chronic hepatitis B infection.

 Thus the title text “ *a patient with uncomplicated chronicity” * is misleading.

 The abstract text “ *from a patient with chronic HBV infection*” is misleading.

 The introduction text “ *HBV chronic strain of subgenotype C2*” is misleading as there is no laboratory proof that the patient had chronic hepatitis B infection

 The methods text “ *The patient was diagnosed with chronic HBV infection*” is misleading as there is no laboratory proof that the patient had chronic hepatitis B infection

 Results and discussion text “ *The patient was diagnosed with chronic HBV infection*” is also misleading the result present show the patient had chronic hepatitis And an HBV infection and that is all without laboratory proof that the patient had chronic hepatitis B infection.

 Also “ *current genomic substitutions of HBV/C2 and to decide treatments against chronic HBV infections”* is again misleading as there is no laboratory proof that the patient had chronic hepatitis B infection

 There is a further statement in the introduction “ *Most of the HBV cases are chronic”* that is not factually correct – most cases of vertical transmission of HBV result in chronic infection, most cases of post-natal acquired HBV infection result in clearance of HBV surface antigen. All cases of HBV infection result in virus latency in the form of HBV cccDNA in liver.

I have read this submission. I believe that I have an appropriate level of expertise to confirm that it is of an acceptable scientific standard, however I have significant reservations, as outlined above.

# Response

**Published:** 2018-09-06

**Authors:** Modhusudon Shaha

**Affiliations:** National Institute of Biotechnology, Bangladesh

We would like to thank the reviewer again for his constructive comments on the manuscript. 

 The manuscript is revised considering the comments and suggestions of the reviewer.

# Peer review: Referee response for version 1

**DOI:** 10.5256/f1000research.16434.r36692

**Published:** 2018-08-07

**Authors:** Paul Klapper

**Affiliations:** 1 University of Manchester, Manchester, UK

This paper reports a sequence analysis of a strain of hepatitis B virus. However, there are some aspects of the paper that merit attention. In the Abstract and again in the Introduction the authors state: *The number of chronic cases of hepatitis B virus (HBV) is increasing rapidly in the world". * I found this an interesting statement and wondered what was the evidence base for this. In the Introduction the authors cite MacLachlan and Cowie (2015). It appears to me that the authors are mis-quoting this reference. The reference actually says "T *he burden of chronic HBV infection is increasingly being recognized*", this is substantially different from suggesting that the number of cases is increasing. There is a general lack of comprehensive epidemiological information on chronic hepatitis B infection as many developing countries (the epicentre of chronic HBV infections) lack surveillance to provide data. We do not know how WHO programmes to prevent vertical transmission of HBV are impacting on chronic hepatitis B and I believe we lack information to support the assertion of a global increase in numbers.

 Also in the abstract - the third line "" *with current common amino acid substitutions" * is not a meaninglful statement. The sentence needs rewriting to make clear what the authors actually mean.

 Introduction 1st paragraph

 last 2 lines: " *which makes it relatively higher risk than other infectious diseases*" ; it is unclear what is meant here, a rate of 2-6% is clearly low compared with risk of, for example, influenza or rotavirus infection.

 Introduction, 2nd Paragraph " *the mutation rate of hepatitis B is high"  and " hence, recombinant strains are evolving with a common pattern". *Mutation is a random event how can a random event lead to a commonly evolving pattern. The authors need to recast the sentence to explain what they really mean.

 Introduction, end of 2nd paragraph. the genome was isolated from " *a patient without liver complication" *yet in methods we are told the patient has chronic liver disease as adjudged by ultrasonography.

 Methods: line 4. Was formal staging not used to describe liver disease e.g. relating shear wave elastography to fibrosis score? Were liver function tests performed?

 Methods, line 5 " *The patient was diagnosed with chronic hepatitis B recently*". This seems strange, the standard definition of chronic hepatitis B infection is the detection of hepatitis B surface antigen in serum for more than 6 months. This patient - with blood taken only one month post diagnosis would not seem to meet the definition. No hepatitis B markers results  are given for the patient and so understanding of the phase of chronic illness (see EASL guidance;Journal of Hepatology 2017 vol. 67: 370–398) is not possible

 Analysis - reference needed for the HBV Geno2Pheno software used.

 What evidence is there that the HBV/C2 isolate sequenced is a common phenotype in chronic hepatitis B virus infection in Bangladesh? Without such evidence it is difficult to see how the conclusions " *The findings of this study will help....*" are justified. This could simply be an single instance of this virus produced through random mutation.It is therefore also difficult to understand how this could be considered a 'reference strain' for chronic hepatitis B as it may represent a single instance and further as the patient does not appear to meet a case definition of having chronic hepatitis B infection, can it be considered as a reference strain for chronic hepatitis B infection.

I have read this submission. I believe that I have an appropriate level of expertise to state that I do not consider it to be of an acceptable scientific standard, for reasons outlined above.

# Response

**Published:** 2018-08-08

**Authors:** Modhusudon Shaha

**Affiliations:** National Institute of Biotechnology, Bangladesh

We would like to thank the reviewer for his constructive comments on the manuscript. Herein, the responses to the comments are given.

*‘The number of chronic cases of hepatitis B virus (HBV) is increasing rapidly in the world’*- The sentence is corrected in the revised manuscript.

*“with current common amino acid substitutions”*- this portion of the sentence is removed from the revised manuscript.

*"which makes it relatively higher risk than other infectious diseases"*- the sentence is re-written with substantial clarity.

*"the mutation rate of hepatitis B is high"  and " hence, recombinant strains are evolving with a common pattern"*- the sentence is re-written in the revised manuscript.

*"a patient without liver complication"*- the sentence is corrected in the revised manuscript.

 Particular liver function tests were not performed. However, the liver was observed normal using ultrasonography.

*"The patient was diagnosed with chronic hepatitis B recently"*- The sentence is edited in the revised manuscript. The detail of diagnosis of the chronicity is described in the revised manuscript.

 Reference is given that study used the HBV Geno2Pheno tool.

*"The findings of this study will help...."*- the sentence is re-written in the revised manuscript.

# Peer review: Referee response for version 1

**DOI:** 10.5256/f1000research.16434.r35849

**Published:** 2018-07-23

**Authors:** Mohammad Ariful Islam

**Affiliations:** 1 Jagannath University, Dhaka, Bangladesh

The overall level of the paper is good: even if it is quite simple, it is well written and some important considerations are highlighted.

 The manuscript talks about the complete genome analysis of hepatitis B virus and represent the complete genome sequence of HBV subgenotype C2 in Bangladesh.

 The study is a short research note. Although the findings of the study is limited and like a genome announcement, it signifies to be documented. The study is scientifically acceptable.

 I have some minor queries

- what does the isolate NHB17965 indicates?
- any reference sequences used in this study? If used, what are these?”

I have read this submission. I believe that I have an appropriate level of expertise to confirm that it is of an acceptable scientific standard.

# Response

**Published:** 2018-08-01

**Authors:** Modhusudon Shaha

**Affiliations:** National Institute of Biotechnology, Bangladesh

We would like to thank the reviewer for his constructive comments on the manuscript.

NHB17965 indicates the sample identification number given by the laboratory. 

There are no reference sequences used in this study for the analysis. The isolated sequence was analyzed using NCBI Genotyping tool and Geno2Pheno tools as given in the manuscript.
